# Characterising the extent of misreporting of high blood pressure, high cholesterol, and diabetes using the Australian Health Survey

**DOI:** 10.1186/s12889-016-3389-y

**Published:** 2016-08-02

**Authors:** Karen Louise Peterson, Jane Philippa Jacobs, Steven Allender, Laura Veronica Alston, Melanie Nichols

**Affiliations:** WHO Collaborating Centre for Obesity Prevention, Faculty of Health, Deakin University, Locked Bag 20001, Geelong, VIC 3220 Australia

**Keywords:** Cardiovascular disease/epidemiology, Diabetes mellitus/epidemiology, Health surveys, Hypertension/epidemiology, Hypercholesterolemia/epidemiology, Logistic models, Multivariate analysis, Odds ratio, Self disclosure

## Abstract

**Background:**

Measuring and monitoring the true prevalence of risk factors for chronic conditions is essential for evidence-based policy and health service planning. Understanding the prevalence of risk factors for cardiovascular disease (CVD) in Australia relies heavily on self-report measures from surveys, such as the triennial National Health Survey. However, international evidence suggests that self-reported data may substantially underestimate actual risk factor prevalence. This study sought to characterise the extent of misreporting in a large, nationally-representative health survey that included objective measures of clinical risk factors for CVD.

**Methods:**

This study employed a cross-sectional analysis of 7269 adults aged 18 years and over who provided fasting blood samples as part of the 2011–12 Australian Health Survey. Self-reported prevalence of high blood pressure, high cholesterol and diabetes was compared to measured prevalence, and univariate and multivariate logistic regression analyses identified socio-demographic characteristics associated with underreporting for each risk factor.

**Results:**

Approximately 16 % of the total sample underreported high blood pressure (measured to be at high risk but didn’t report a diagnosis), 33 % underreported high cholesterol, and 1.3 % underreported diabetes. Among those measured to be at high risk, 68 % did not report a diagnosis for high blood pressure, nor did 89 % of people with high cholesterol and 29 % of people with high fasting plasma glucose. Younger age was associated with underreporting high blood pressure and high cholesterol, while lower area-level disadvantage and higher income were associated with underreporting diabetes.

**Conclusions:**

Underreporting has important implications for CVD risk factor surveillance, policy planning and decisions, and clinical best-practice guidelines. This analysis highlights concerns about the reach of primary prevention efforts in certain groups and implications for patients who may be unaware of their disease risk status.

## Background

Effective and appropriate health system planning and evaluation relies on accurate estimates of disease prevalence. Cardiovascular disease (CVD) and diabetes represent major causes of ill health in Australia and have been National Health Priority Areas since the 1990s [1, 2]. CVD is a leading cause of death in Australia, accounting for 13.9 % of the burden of disease in 2010, while diabetes mellitus accounted for a further 2.3 % [3]. A substantial proportion of healthcare expenditure in Australia is directed toward CVD and diabetes, with $7.6 billion (around 12 %) and $1.5 billion (2.3 %) of 2008–09 expenditure attributable to cardiovascular diseases and diabetes, respectively [4, 5].

Population estimates of chronic disease and associated risk factor rates are commonly based on self-report data obtained via interview or questionnaire. Self-report data can be obtained readily and at minimal expense for a large population sample, but may not provide an accurate indication of prevalence [6]. Evidence from several international studies suggests that clinical risk factors are frequently misreported [6–10], with respondents either not reporting risks for which they have biomarkers (underreporting), or reporting having risks for which they lack biomarkers (overreporting). Apparent overreporting may be a result either of inaccurate reporting, or of successful treatment of a condition (for instance, with medication), causing biomedical results to appear normal. Conversely, underreporting may represent unknown and therefore untreated risk at the individual level. For health systems to effectively prevent chronic conditions like CVD and diabetes, risks for and markers of disease must first be detected and reported, to facilitate planning and response. For this reason, underreporting has important implications for surveillance and health system planning and is the focus of this analysis.

Multiple factors may contribute to underreporting; a patient may be unaware of their risk status if they haven’t been diagnosed by a doctor; there may be miscommunication between doctor and patient; or a patient may choose not to report a known diagnosis for various personal reasons [6, 11, 12]. The extent of underreporting can be substantial and varies by risk factor, country and socio-demographic characteristics [6–10, 13, 14]. Risks factors that are rarely screened for and that cause minimal symptoms are more likely to be underreported [6].

Australian estimates of the prevalence of CVD risk factors are largely reliant on self-report measures from surveys such as the triennial National Health Survey (Australian Bureau of Statistics, ABS), and administrative data such as the National Hospital Morbidity Database (Australian Institute of Health and Welfare) and the National Diabetes Services Scheme (Diabetes Australia). It is difficult to validate the prevalence estimates generated by these types of data sources without objective measures such as blood tests, which necessitates a large-scale biomedical survey. Such data became available in Australia for the first time with the large, comprehensive, and nationally-representative 2011–12 Australian Health Survey (AHS).

The research questions informing this study were:

1. What is the extent of misreporting of cardiovascular risk factors in Australia? and,
2. What socio-demographic characteristics are associated with underreporting?

## Methods

### Study sample

This analysis focuses on high blood pressure, high cholesterol, and diabetes, which are all major risk factors for CVD [15, 16]. It uses data from the core sample of the 2011–12 AHS, a nationwide, household survey consisting of three arms: a general health survey, a nutrition and physical activity survey, and a voluntary biomedical survey which included participants from the first two arms. The AHS used a multi-stage, stratified area sample of private residences in both urban and rural areas, covering approximately 97 % of people living in Australia. Out of 30,721 households selected for the sample, 25,084 households (81.6 %) adequately responded. The core sample included 31,837 persons, of whom 24,910 were adults aged 18 years and over [17]. Survey respondents were asked whether they had ever been told by a doctor or nurse that they had “high blood pressure or hypertension,” “high cholesterol,” or “diabetes,” and whether the condition was current and long-term. Trained interviewers took blood pressure readings. All adult core sample respondents were invited to participate in the biomedical survey and 7582 (30.4 %) ultimately provided a fasting blood sample. Detailed information on survey structure, design and sampling can be found in the AHS Users’ Guide [17].

Results were analysed for 7269 non-pregnant adults aged 18 years and over who had valid readings for blood pressure, total cholesterol, and fasting plasma glucose (FPG). High measured blood pressure was defined as a reading greater than or equal to 140/90 mmHg [18]. High measured cholesterol was defined as a total blood cholesterol reading greater than or equal to 5.5 mmol/L [19]. A FPG level greater than or equal to 7.0 mmol/L was considered indicative of diabetes [20]. As FPG tests cannot differentiate types of diabetes, this analysis grouped self-reported type 1 and type 2 diabetes mellitus. Data on treatment for long-term conditions were not available, therefore this study did not include an analysis of whether respondents were being treated for reported conditions.

### Statistical analysis

For each risk factor, self-reported prevalence was tabulated against the relevant biomedical indicator to determine reporting accuracy. A two-proportion z test was used to determine whether any difference between self-reported and measured prevalence was statistically significant. Kappa statistics were generated to indicate the level of agreement between self-reported and measured data. Univariate logistic regression models were used to calculate the odds of underreporting according to socio-demographic characteristics. Multivariate logistic regression models were developed to analyse concurrent associations between different socio-demographic characteristics. Sample weights provided by the ABS for the biomedical survey were applied in order to benchmark the biomedical sample to the 31 October 2011 estimated resident population as described in the AHS User’s Guide [17]. Variables associated with underreporting in univariate analysis at *p* < 0.25 were included in the multivariate model in a forward, step-wise procedure as described by Bursac et al. [21], and were retained in the final adjusted model if they improved the model fit.

### Outcome variables

Underreporting was calculated by dividing the number of people who had biomarkers for a given risk factor but did not report it, by the total number of people with biomarkers for that risk factor. For the purposes of the logistic regression, a binary outcome variable was generated with people who accurately reported disease being coded as 0 and underreporters coded as 1. Overreporting was calculated by dividing the number of people who reported a given risk factor but lacked biomarkers for it, by the total number of people who reported being diagnosed with that risk factor. As underreporting is the main concern of this analysis, overreporting was not included in the logistic regression.

### Socio-demographic variables

Socio-demographic characteristics included sex, age, education (highest year of school completed), equivalised weekly household income, whether born in Australia, self-rated English proficiency, area-level socio-economic status and remoteness. The effect of age on underreporting was analysed in two ways: once with age as a continuous variable, with the odds ratio reported per 1 year increase in age, and once with age divided into three groups (18–44 years old, 45–64 years old, and 65 years and over) to reflect Australian clinical practice guidelines [22], which recommend separate preventive actions in “middle aged” adults (defined as 45–64 years) and “older aged” adults (defined as 65 years and over).

The ABS Index of Relative Socio-economic Disadvantage (IRSD) was used as an indicator of relative area-level socioeconomic status. IRSD summarises a range of census information about the economic and social conditions of people and households within an area [23]. Remoteness was defined according to the ABS Australian Statistical Geographic Standard (ASGS) remoteness classification system, a geographical structure which classifies areas sharing common characteristics of remoteness into five groups: major cities, inner regional, outer regional, remote, and very remote [24]. This analysis differentiated between major cities, inner regional areas, and all other areas (outer regional, remote and very remote). All analyses used expanded confidentialised unit record files of the AHS core sample and were conducted within the ABS’s Remote Access Data Laboratory [25] with queries submitted in Stata 10 analytical language. A *p*-value <0.05 was used as a threshold for determining statistical significance of the multivariate models.

## Results

Approximately 55 % of the sample was female, and the mean age was 51.9 (standard deviation 0.2) years (Table 1). The majority of respondents were in the 45–64 year age group (41 %) and lived in major cities (61 %). Almost half had completed year 12 or equivalent education, while another 40 % had completed only year 10 or below.

**Table 1:** Socio-demographic characteristics of the study sample

	Male (*n* = 3275)	Female (*n* = 3994)	All (*n* = 7269)
	%	%	%
Mean age, years (SD)	52.5 (0.3)	51.4 (0.3)	51.9 (0.2)
Age groups			
18–44	32.8	35.7	34.4
45–64	41.0	40.2	40.6
65+	26.1	24.1	25.0
ASGS remoteness classification			
Major cities	60.8	61.2	61.0
Inner regional areas	22.8	22.6	22.7
Other areas	16.4	16.2	16.3
Equivalised household income			
Quintile 1 (lowest income)	16.1	19.5	18.0
Quintile 2	18.1	21.0	19.7
Quintile 3	19.9	19.6	19.7
Quintile 4	22.2	21.3	21.7
Quintile 5 (highest income)	23.7	18.6	20.9
IRSD (area-level disadvantage)			
Quintile 1 (highest disadvantage)	18.1	18.2	18.1
Quintile 2	19.3	20.4	19.9
Quintile 3	20.9	20.6	20.7
Quintile 4	20.8	20.1	20.4
Quintile 5 (lowest disadvantage)	20.9	20.7	20.8
Highest year of school completed			
Year 9 or below	16.4	15.2	15.8
Year 10 or equivalent	24.6	24.6	24.6
Year 11 or equivalent	10.0	9.5	9.7
Year 12 or equivalent	49.0	50.7	49.9
Region of birth			
Australia	69.9	72.3	71.2
Northwest Europe	12.7	10.6	11.6
Oceania and Antarctica (excluding Australia)	3.5	3.3	3.4
Southeast Asia	2.7	3.5	3.2
Southern and Eastern Europe	2.7	3.0	2.9
Southern and Central Asia	2.6	2.0	2.3
Sub-Saharan Africa	1.6	1.5	1.6
Northeast Asia	1.6	1.5	1.6
Americas	1.5	1.3	1.4
North Africa and Middle East	1.1	0.9	1.0
English proficiency			
Very well or mainly speaks English at home	95.6	95.2	95.4
Well	3.3	3.4	3.4
Not well or not at all	1.1	1.4	1.2

Notes: Columns may not sum to 100 % due to rounding artefacts. *ASGS* Australian Statistical Geography Standard, *IRSD* Index of Relative Socio-economic Disadvantage, *SD* standard deviation

### Self-reported and measured prevalence of risk factors

**Fig. 1:**
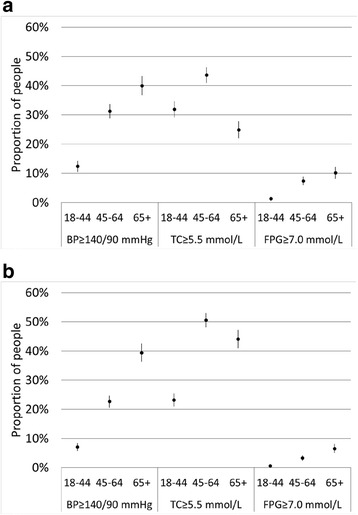
Total population prevalence of measured high blood pressure, high total cholesterol, and elevated fasting plasma glucose, by sex and age group. **a** Males. **b** Females. Notes: *BP* blood pressure, *TC* total serum cholesterol, *FPG* fasting plasma glucose

Self-reported prevalence was lower than measured prevalence for high blood pressure (17.4 and 23.9 %, respectively) and high cholesterol (12.2 and 37.3 %, respectively) (Table 2). For diabetes, 6.1 % of people self-reported having diabetes, but only 4.5 % were measured to have elevated blood glucose levels. The difference between self-reported and measured prevalence was statistically significant for both sexes for all three risk factors. Figure 1 presents the measured prevalence in (a) males and (b) females, by age group. The prevalence of high blood pressure and diabetes increased with age for both sexes, while high cholesterol was most prevalent in the 45–64 year age group.

**Table 2:** Prevalence of risk factors, accuracy of reporting, and rates of misreporting

		High blood pressure	High cholesterol	Diabetes
Prevalence	Self-reported	17.4 (16.5–18.3)	12.2 (11.5–13.0)	6.1 (5.6–6.7)
	Measured to have risk factor	23.9 (22.9–24.9)*	37.3 (36.2–38.4)*	4.5 (4.0–4.9)*
Accuracy of self-report	Overreported: Self-reported but no measured risk factor	9.8 (9.1–10.5)	8.1 (7.5–8.8)	2.9 (2.6–3.3)
	Underreported: Measured to have risk factor but did not self-report	16.4 (15.5–17.2)	33.2 (32.1–34.3)	1.3 (1.0–1.5)
	Accurate, has disease: Self-reported and measured to have risk factor	7.6 (7.0–8.2)	4.1 (3.6–4.5)	3.2 (2.8–3.6)
	Accurate, does not have disease: Did not self-report and no measured risk factor	66.3 (65.2–67.3)	54.6 (53.4–55.7)	92.6 (92.0–93.2)
Misreporting rate	Proportion of those measured to have risk factor that did not self-report	68.4 (66.2–70.6)	89.0 (87.9–90.2)	28.6 (23.7–33.6)
	Proportion of those who self-reported that had no measured risk factor	56.5 (53.8–59.2)	66.6 (63.5–69.7)	48.0 (43.3–52.6)

Notes: Proportion of people (95 % confidence intervals in brackets)

*The difference between self-reported and measured prevalence was statistically significant (*p* < 0.001)

### Misreporting

**Fig. 2:**
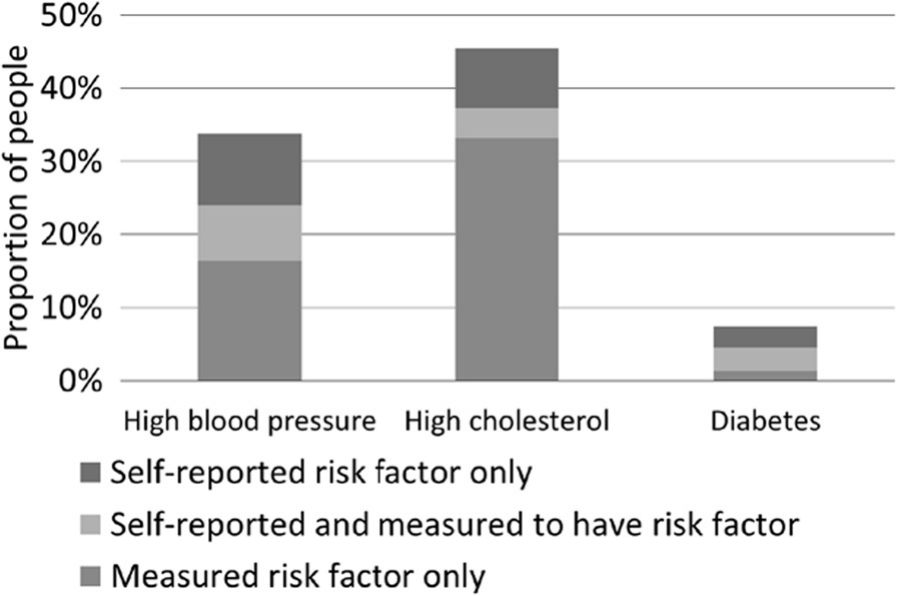
Prevalence of overreporting, accurate reporting, and underreporting, by risk factor

While the majority of people were correct about not having a given risk factor, both underreporting and overreporting were present for all three risk factors (Table 2). Just under 8 % of people had high blood pressure and accurately reported it, while 4.1 and 3.2 % accurately reported high cholesterol and diabetes, respectively. Figure 2 gives a graphical representation of the amount of overlap between self-reported and measured risk factors. Participants measured to have risk factors were often not the same people who self-reported having risk factors, especially for high cholesterol, indicating that the extent of misreporting at the individual level was greater than the overall differences between self-report and measured prevalence would suggest. Kappa statistics were calculated to measure the agreement between self-reported and measured data, and were 0.21 (95 % CI: 0.18–0.23) for high blood pressure and −0.02 (−0.04--0.01) for high cholesterol, indicating low agreement, and 0.58 (0.54–0.62) for diabetes, indicating moderate agreement using the scale recommended by Landis and Koch (1977) [26].

Approximately 16.4 % of all respondents underreported high blood pressure, 33.2 % underreported high cholesterol, and 1.3 % underreported diabetes. Among those measured to have each risk factor, a large proportion did not self-report (Table 2). The proportion of people with high measured blood pressure who failed to report it was 68.4 % (66.2–70.6 %). Of those with high measured total cholesterol, 89.0 % (87.9–90.2 %) did not report a diagnosis of high cholesterol. Of people with elevated FPG, 28.6 % (23.7–33.6 %) did not report a diagnosis of diabetes. On the other hand, of those who self-reported high blood pressure and high cholesterol, the majority did not have biomarkers (56.5 % overreported high blood pressure and 66.6 % overreported high cholesterol). Almost half of those who self-reported diabetes (48.0 %) did not have FPG levels indicating diabetes.

### Socio-demographic factors associated with underreporting

Univariate logistic regression analysis showed that the older age groups had significantly lower odds of underreporting high blood pressure than the 18–44 age group, with an odds ratio in the 45–64 year age group of 0.4 (95 % CI 0.2–0.6) and in the 65 and over age group of 0.2 (0.1–0.3) (Table 3). When age was treated as a continuous variable, the odds ratio for underreporting corresponding to each full-year increase in age from 18 years was 0.96 (0.95–0.97). Higher education level was associated with greater underreporting of high blood pressure; the odds of underreporting in the highest education group (finished year 12 or above) were 1.7 (1.2–2.5) times higher than in those who had finished only year 9 or below. In the group who finished year 11 or below, the odds were 2.3 (1.4–4.0) times higher than the lowest education group. Higher equivalised household income was also associated with greater underreporting of high blood pressure, with an odds ratio of 1.9 (1.2–3.1) in the second highest and 2.4 (1.5–3.8) in the highest income group compared to the lowest income group. However, household income was found to be correlated with age (r_S_ < −0.34), education (r_S_ < −0.37), and area-level disadvantage (r_S_ > 0.32), and its inclusion in the multivariate analysis did not improve the fit of the final model, therefore it was excluded from the final multivariate model. In the fully-adjusted multivariate logistic regression model for high blood pressure, age remained significantly associated with underreporting (Table 4). No other variables remained significant, though the overall model, which also included sex and education, was significant (*p* < 0.001).

**Table 3:** Univariate logistic regressions of factors associated with underreporting

	High blood pressure		High cholesterol		Diabetes	
	OR	95 % CI	OR	95 % CI	OR	95 % CI
Sex						
Male	1.0 (ref)		1.0 (ref)		1.0 (ref)	
Female	0.9	(0.7–1.2)	1.1	(0.8–1.6)	0.5	(0.2–1.0)*
Age (per year increase in age from age 18)	0.96	(0.95–0.97)**	0.98	(0.97–0.99)**	0.99	(0.96–1.02)
Age group						
18–44 years	1.0 (ref)		1.0 (ref)		1.0 (ref)	
45–64 years	0.4	(0.2–0.6)**	0.5	(0.3–0.7)**	1.2	(0.4–4.0)
65+	0.2	(0.1–0.3)**	0.5	(0.3–0.8)**	0.8	(0.2–2.5)
ASGS remoteness classification						
Major cities	1.0 (ref)		1.0 (ref)		1.0 (ref)	
Inner regional areas	0.9	(0.6–1.3)	1.0	(0.6–1.4)	1.0	(0.4–2.5)
Other areas	1.0	(0.6–1.5)	1.1	(0.6–1.8)	0.7	(0.3–1.7)
Equivalised household income						
Quintile 1 (lowest income)	1.0 (ref)		1.0 (ref)		1.0 (ref)	
Quintile 2	1.0	(0.7–1.5)	0.9	(0.5–1.6)	1.0	(0.4–2.6)
Quintile 3	1.6	(1.0–2.6)	0.8	(0.5–1.4)	4.1	(1.5–10.8)**
Quintile 4	1.9	(1.2–3.1)**	1.2	(0.7–2.0)	2.9	(1.0–8.5)
Quintile 5 (highest income)	2.4	(1.5–3.8)**	1.0	(0.6–1.7)	3.4	(1.1–10.7)*
IRSD (area-level disadvantage)						
Quintile 1 (highest disadvantage)	1.0 (ref)		1.0 (ref)		1.0 (ref)	
Quintile 2	0.9	(0.6–1.5)	1.0	(0.6–1.8)	2.5	(0.8–7.8)
Quintile 3	1.0	(0.6–1.5)	1.3	(0.7–2.3)	1.0	(0.3–3.3)
Quintile 4	0.9	(0.6–1.5)	0.9	(0.5–1.6)	2.8	(0.9–8.3)
Quintile 5 (lowest disadvantage)	1.4	(0.8–2.2)	0.9	(0.5–1.7)	4.1	(1.4–12.3)*
Education level						
Year 9 or below	1.0 (ref)		1.0 (ref)		1.0 (ref)	
Year 10 or equivalent	1.2	(0.8–1.8)	1.3	(0.8–2.1)	1.4	(0.5–3.8)
Year 11 or equivalent	2.3	(1.4–4.0)**	1.5	(0.8–2.7)	1.8	(0.5–6.0)
Year 12 or equivalent	1.7	(1.2–2.5)**	1.8	(1.2–2.9)**	1.0	(0.4–2.7)
English Proficiency						
Fluent	1.0 (ref)		1.0 (ref)		1.0 (ref)	
Not fluent	1.2	(0.6–2.5)	0.7	(0.3–1.3)	2.7	(0.7–10.1)
Country of birth						
Australia	1.0 (ref)		1.0 (ref)		1.0 (ref)	
Not Australia	1.2	(0.9–1.6)	1.0	(0.7–1.4)	1.1	(0.5–2.3)

Notes: *CI* 95 % confidence interval, *IRSD* Index of Relative Socio-economic Disadvantage, *OR* odds ratio, *ref* reference group

*Group significantly different from reference group (*p* < 0.05)

**Group significantly different from reference group (*p* < 0.01)

**Table 4:** Multivariate logistic regressions of factors associated with underreporting

	High blood pressure		High cholesterol		Diabetes	
	OR	95 % CI	OR	95 % CI	OR	95 % CI
Sex						
Male	1.0 (ref)		1.0 (ref)		1.0 (ref)	
Female	1.1	(0.8–1.4)	1.3	(0.9–1.8)	0.6	(0.3–1.3)
Age group						
18–44 years	1.0 (ref)		1.0 (ref)		Not in model	
45–64 years	0.4	(0.2–0.6)***	0.5	(0.3–0.7)**		
65+	0.2	(0.1–0.3)***	0.5	(0.3–0.8)*		
Equivalised household income						
Quintile 1 (lowest income)	Not in model		Not in model		1.0 (ref)	
Quintile 2					1.0	(0.4–2.7)
Quintile 3					3.7	(1.3–10.7)*
Quintile 4					2.4	(0.8–7.1)
Quintile 5 (highest income)					2.7	(0.8–8.8)
IRSD (area-level disadvantage)						
Quintile 1 (highest disadvantage)	Not in model		Not in model		1.0 (ref)	
Quintile 2					1.9	(0.7–5.6)
Quintile 3					0.8	(0.3–2.7)
Quintile 4					2.2	(0.7–7.1)
Quintile 5 (lowest disadvantage)					3.7	(1.3–11.0)*
Education level						
Year 9 or below	1.0 (ref)		1.0 (ref)		Not in model	
Year 10 or equivalent	0.9	(0.6–1.4)	1.1	(0.7–1.8)		
Year 11 or equivalent	1.7	(1.0–3.0)	1.3	(0.7–2.3)		
Year 12 or equivalent	1.0	(0.7–1.6)	1.5	(0.9–2.3)		
English Proficiency						
Fluent	Not in model		1.0 (ref)		1.0 (ref)	
Not fluent			0.6	(0.3–1.1)	3.6	(0.9–14.5)
*p*-value of model	*p* < 0.001		*p* < 0.01		*p* < 0.001	

Notes: *CI* confidence interval, *IRSD* Index of Relative Socio-economic Disadvantage, *OR* odds ratio, *ref* reference group

*Group significantly different from reference group (*p* < 0.05)

**Group significantly different from reference group (*p* < 0.01)

***Group significantly different from reference group (*p* < 0.001)

Older age was also associated with lower odds of underreporting high cholesterol. The odds of underreporting high cholesterol in both the 45–64 and the 65 and over age groups was half that of the 18–44 year age group (45–64 years: 0.5 (0.3–0.7); 65 years and over: 0.5 (0.3–0.8)). When treated as a continuous variable, each year increase in age was associated with an odds ratio of 0.98 (0.97–0.99). Higher education level was associated with increased odds of underreporting high cholesterol. People who finished year 12 or above had 1.8 (1.2–2.9) times the odds of underreporting when compared to people who had only finished year 9 or below. In the fully-adjusted model, however, only being in the youngest age group remained significantly associated with underreporting, though the model was significant to the *p* < 0.01 level with education, sex and English proficiency also accounted for.

Male sex, higher household income and lower area-level disadvantage were significantly associated with underreporting diabetes in univariate analysis, but age was not. The odds of underreporting were 50 % lower among females compared to males (0.5 (0.2–1.0)). The odds of underreporting were 3.4 (1.1–10.7) times higher in the highest household income quintile versus the lowest quintile. Likewise, people living in areas with the lowest disadvantage (IRSD quintile 5) had 4.1 (1.4–12.3) times the odds of underreporting diabetes compared to those living in the most disadvantaged areas (IRSD quintile 1). Though household income was collinear with IRSD, the multivariate model had a better fit when both variables were included. Household income quintile 3 was significantly associated with greater odds of underreporting (3.7 (1.3–10.7)), as was IRSD quintile 5 (3.7 (1.3–11.0)). Sex and English proficiency were also in the model, but were not significant.

## Discussion

In this representative sample of Australian adults, both underreporting and overreporting were prevalent for all three risk factors. The combined effect of underreporting and overreporting was such that the self-reported prevalence of high blood pressure, high cholesterol, and diabetes did not appear to be vastly different from the measured prevalence, yet there was minimal overlap between people reporting risk factors and those measured to have them. Furthermore, kappa statistics indicated low agreement between self-reported and measured data. Both high blood pressure and high cholesterol were highly prevalent in the study population, and rates of under and overreporting were comparable for both, as were the characteristics of those most likely to underreport each risk factor. Approximately 16.4 % of the total sample had high blood pressure but didn’t report it, 33.2 % failed to report high cholesterol, and 1.3 % failed to report diabetes. The majority of people measured to have high blood pressure and high cholesterol did not report any diagnosis, while nearly one-third of those with impaired FPG did not report diabetes. Underreporting was most prevalent in those with higher SES (for diabetes) and in younger adults (for blood pressure and cholesterol).

Underreporting could be due to any of several factors. The individual may not experience symptoms and may not seek medical care; their doctor may not screen for a risk factor or may decide not to diagnose; a diagnosis may be miscommunicated or forgotten; or an individual may choose not to report for various personal reasons [6, 11, 12]. People from more disadvantaged areas and lower income households had lower odds of underreporting diabetes, contrary to expected results. People of higher socio-economic status (SES) could reasonably be expected to have greater access to resources, more frequent contact with the health system, and better rates of diagnosis. Higher underreporting in people of higher SES may be evidence of a social desirability bias, wherein people of higher SES feel more compelled to conform to a ‘healthy norm.’ This phenomenon has been demonstrated in many survey situations [6], but is yet to be validated within the Australian context.

Older adults were significantly more likely to report high blood pressure or high cholesterol than were younger adults, despite both risk factors being prevalent in the 18–44 year age group (Fig. 1). One potential explanation could be that younger adults have less contact with the health system than older adults, a phenomenon documented in the US [27] and Australia [28]. Young adults may not feel unwell or expect to be suffering from chronic diseases that are more commonly associated with older age, and therefore may not seek care. High blood pressure and high cholesterol increase the risk of atherosclerosis, but the effects of atherosclerotic disease accrue gradually over one’s lifetime and may not cause symptoms in younger adults [29]. The young may also be less likely to be tested for chronic disease risk factors when they do contact the health system [30, 31]. Data from the US have shown substantially lower rates of awareness, treatment and control of hypertension in adults aged 18–39 years with high blood pressure versus those aged 40 years and over [32].

Underreporting was particularly pronounced for high cholesterol, with nearly 90 % of people with high serum cholesterol levels not reporting any diagnosis, and younger adults had significantly lower odds of reporting compared to older adults. In Australia, regular cholesterol tests are currently only recommended for people aged 45 years and over [22]; it is likely that people in the younger age group did not have cholesterol levels regularly tested. Primary prevention of disease relies on early detection of risk in order to halt progress from risk to disease. The measured prevalence of high cholesterol was 32 % in the 18–44 age group, representing risk for atherosclerosis later in life, yet few of these people reported being aware that they had this risk factor. This result suggests that it may be appropriate to review clinical guidelines and to consider recommending that screening for high cholesterol begins earlier in adulthood. Furthermore, underreporting was exceptionally high in all adults aged 45 and over who had high measured cholesterol, suggesting that even with targeted clinical practice guidelines, the large majority of adults remain unaware of their risk status.

Underreporting likely represents risk in the population that is not being detected by the health system, with implications for individuals and their families, as well as for the wider health system. The data collected within the AHS do not allow for in-depth analysis into which factors might have influenced misreporting in this sample, but it is likely that the underlying causes will differ by socio-demographic factors. Non-uniform rates of misreporting have significant implications for health promotion efforts and policy decisions, particularly those aimed at reducing health inequalities. Misreporting also has implications for health system planning, which relies on accurate estimates of current and future burden of disease.

### Strengths and limitations

While the 2011–12 AHS had a high household response rate (81.6 %) [17] and is nationally-representative, the sample for this analysis was limited to the voluntary biomedical component. A biomedical sample weight was applied to the logistic regression analysis to account for the complex sampling design. However, it is possible that a response bias was present in the study subsample when compared to the core sample. Indeed, only 30.4 % of invited adults provided fasting blood samples. However, a comparison between the biomedical subsample and the core sample (available in the AHS users’ guide [17]) shows that participants in the biomedical component did not differ greatly from the wider sample with respect to measured demographic characteristics and risk factor profiles.

Some aspects of this analysis were limited by the breadth of data collected in the original survey. Respondents were asked to report any current, long-term conditions and were prompted for specific conditions, but were not provided with definitions or clarification for each condition. This may lead to misreporting if respondents were unsure whether a diagnosis qualified to be reported (for example, a patient with a systolic blood pressure of 130 being told their blood pressure is on the high side may or may not think this qualifies as “high blood pressure”).

Further, the analysis was limited by a lack of treatment (medication) information for the full, biomedical sample. For this reason, data on apparent overreporting, while included here for completeness, must be interpreted with caution. Participants who reported risk factors but lacked biomarkers did not necessarily ‘misreport’ or ‘overreport’. They may well have been diagnosed with the risk factor by a health professional, despite biomarkers being currently absent. The inability to take into account whether respondents were on medication for the included risk factors could lead to apparent overreporting if medication is effectively keeping patients’ biomarker levels below clinical thresholds. Other healthy lifestyle changes following a diagnosis could also have improved biomarker levels. Despite these concerns, measured biomarker levels represent the best ‘gold standard’ available for determining the validity and accuracy of self-report data for chronic conditions [6]. Indeed, the use of objective, biomedical data as a comparator of self-reported data is a key strength of this study. Many studies compare self-reports to hospital or physician records, which may be subject to various additional types of bias or error [33]. Additionally, this study used a large and nationally-representative sample, further strengthening the results, and is the first such analysis of CVD risk factors to be conducted in Australia.

It is well documented that Australians in regional and remote areas suffer limited access to health services compared to their urban counterparts [34] and therefore might be expected to be less aware of their risk status; however, this study did not show any association between underreporting and remoteness. This finding may warrant further investigation, as despite known limitations in health care access, the result suggests that the services available and accessed may be having at least a comparable impact to that offered in major cities.

The prevalence rates and rates of underreporting found in this study were comparable to those reported in previous studies in a variety of international settings, though most have focused on elderly populations. The measured prevalence rates of each risk factor observed in this study were similar to, though higher than, those reported in another Australian study exploring risk factors in a workplace environment [35]. While the measured prevalence rates of high blood pressure, high cholesterol, and impaired FPG in the current study were 24, 37, and 4.5 %, respectively, Freak-Poli et al. (2010) reported prevalence rates of 18, 28, and 2 %. However, there were important differences between the sample characteristics; the sample in the Freak-Poli study was an average of 12 years younger than the AHS sample used for this study, was more highly educated (80 % with tertiary education versus only 50 % completing year 12 or more), and 100 % were employed (versus 67 % in the AHS sample). Similar to previous studies in Taiwan and Denmark, the rate of misreporting in the current analysis varied depending on the risk factor [7, 8]. Measured prevalence rates of high blood pressure have been demonstrated to be 7–20 percentage points higher than self-reported rates [7, 8, 12, 13, 36, 37]. Reporting of high cholesterol has been less frequently validated, but differences in the order of 30 percentage points between self-reported and measured population prevalence were demonstrated in the US [12, 36]. Diabetes self-reported and measured prevalence rates are often within one percentage point of each other, similar to the results found here [6–8].

Misreporting, and particularly underreporting, are of great concern from an individual, clinical and public health perspective. While self-reported measures are easy to collect, this analysis indicates that they may be failing to provide an accurate picture of the risk and burden of CVD in Australia, which has implications for surveillance and policy planning and decisions. This analysis raises questions about the effectiveness and reach of primary prevention efforts in certain demographic groups and potential implications for patients who may be unaware of their disease risk status. The relationships between both age and socio-economic indicators and underreporting should be further explored to identify the reasons for high levels of underreporting in particular demographic groups.

## Conclusions

This study found a substantial amount of misreporting of high blood pressure and high cholesterol, and to a lesser extent, of diabetes. Health system planning, both in the short and long-term, relies on effective surveillance of risk and disease in the population. Inaccurate estimates of prevalence could lead to shortages of resources or overspending. Furthermore, failure to provide primary or secondary prevention and care could place substantial sections of the population at risk of developing heart disease and diabetes complications, and subsequent loss of quality of life.

## Abbreviations

ABS, Australian Bureau of Statistics; AHS, Australian Health Survey; ASGS, Australian Statistical Geographic Standard; BP, blood pressure; CI, confidence interval (95 %); CVD, cardiovascular disease; FPG, fasting plasma glucose; IRSD, Index of Relative Socio-economic Disadvantage; OR, odds ratio; ref, reference group; SD, standard deviation; SES, socio-economic status; TC, total cholesterol; US, United States

## References

[1] Australian Institute of Health and Welfare. Cardiovascular disease, diabetes and chronic kidney disease–Australian facts: Prevalence and incidence. Cardiovascular, diabetes and chronic kidney disease series. 2014. http://www.aihw.gov.au/WorkArea/DownloadAsset.aspx?id=60129549614. Accessed 09 Mar 2016

[2] Australian Institute of Health and Welfare. National health priority areas. 2015. http://www.aihw.gov.au/national-health-priority-areas/. Accessed 11 Sep 2015

[3] GBD Compare. Institute for Health Metrics and Evaluation, Seattle, WA. 2013. http://ihmeuw.org/3c4a. Accessed 01 Jun 2015

[4] Australian Institute of Health and Welfare. Health-care expenditure on cardiovascular diseases 2008–09. CVD 65. 2014. http://www.aihw.gov.au/WorkArea/DownloadAsset.aspx?id=60129546379. Accessed 09 Mar 2016

[5] Australian Institute of Health and Welfare. Diabetes expenditure in Australia 2008–09. Cat no CVD 62. 2013. http://www.aihw.gov.au/WorkArea/DownloadAsset.aspx?id=60129543916. Accessed 09 Mar 2016

[6] Newell SA, Girgis A, Sanson-Fisher RW, Savolainen NJ (1999). The accuracy of self-reported health behaviors and risk factors relating to cancer and cardiovascular disease in the general population: a critical review. Am J Prev Med.

[7] Goldman N, Lin IF, Weinstein M, Lin YH (2003). Evaluating the quality of self-reports of hypertension and diabetes. J Clin Epidemiol.

[8] Frost M, Wraae K, Gudex C, Nielsen T, Brixen K, Hagen C (2012). Chronic diseases in elderly men: underreporting and underdiagnosis. Age Ageing.

[9] Kriegsman DMW, Penninx BWJH, Van Eijk JTM, Boeke AJP, Deeg DJH (1996). Self-reports and general practitioner information on the presence of chronic diseases in community dwelling elderly: a study on the accuracy of patients’ self-reports and on determinants of inaccuracy. J Clin Epidemiol.

[10] Okura Y, Urban LH, Mahoney DW, Jacobsen SJ, Rodeheffer RJ (2004). Agreement between self-report questionnaires and medical record data was substantial for diabetes, hypertension, myocardial infarction and stroke but not for heart failure. J Clin Epidemiol.

[11] O’Brien K. Living dangerously: Australians with multiple risk factors for cardiovascular disease. Bulletin 24 AIHW Cat no AUS 57. 2005. http://www.aihw.gov.au/publication-detail/?id=6442467692. Accessed 08 Mar 2016

[12] Bowlin SJ, Morrill BD, Nafziger AN, Jenkins PL, Lewis C, Pearson TA (1993). Validity of cardiovascular disease risk factors assessed by telephone survey: the Behavioral Risk Factor Survey. J Clin Epidemiol.

[13] Mentz G, Schulz AJ, Mukherjee B, Ragunathan TE, Perkins DW, Israel BA (2012). Hypertension: development of a prediction model to adjust self-reported hypertension prevalence at the community level. BMC Health Serv Res.

[14] Vargas CM, Burt VL, Gillum RF, Pamuk ER (1997). Validity of self-reported hypertension in the National Health and Nutrition Examination Survey III, 1988–1991. Prev Med.

[15] World Heart Federation. Hypertension. 2015. http://www.world-heart-federation.org/cardiovascular-health/cardiovascular-disease-risk-factors/hypertension/. Accessed 24 Mar 2015

[16] World Heart Federation. Cardiovascular disease risk factors. 2015. http://www.world-heart-federation.org/cardiovascular-health/cardiovascular-disease-risk-factors/. Accessed 24 Mar 2015

[17] Australian Bureau of Statistics. 4363.0.55.001–Australian Health Survey: Users’ Guide, 2011–13. 2013. http://www.abs.gov.au/ausstats/abs@.nsf/Latestproducts/4363.0.55.001Main%20Features12011-13?opendocument&tabname=Summary&prodno=4363.0.55.001&issue=2011-13&num=&view=. Accessed 09 Mar 2016

[18] Australian Bureau of Statistics. 4363.0.55.001–Australian Health Survey: Users’ Guide, 2011–13: Blood pressure. 2013. http://www.abs.gov.au/ausstats/abs@.nsf/Latestproducts/78B3C16892876C2ECA257B8D00229E99?opendocument. Accessed 23 Nov 2015

[19] Australian Bureau of Statistics. 4363.0.55.001–Australian Health Survey: Users’ Guide, 2011–13: Total cholesterol. 2013. http://www.abs.gov.au/ausstats/abs@.nsf/Lookup/6B4B5CC93F32E1E1CA257C3D000D8903?opendocument. Accessed 23 Nov 2015

[20] Australian Bureau of Statistics. 4363.0.55.001–Australian Health Survey: Users’ Guide, 2011–13: Diabetes biomarkers. 2013. http://www.abs.gov.au/ausstats/abs@.nsf/Latestproducts/4E3E32BE5981C674CA257C3D000D87DF?opendocument. Accessed 23 Nov 2015

[21] Bursac Z, Gauss CH, Williams DK, Hosmer DW (2008). Purposeful selection of variables in logistic regression. Source Code Biol Med.

[22] Royal Australian College of General Practitioners. Guidelines for preventive activities in general practice. 8th edn. 2012. http://www.racgp.org.au/your-practice/guidelines/redbook/. Accessed 09 Mar 2016

[23] Australian Bureau of Statistics. Socio-Economic Indexes for Areas (SEIFA) 2011–Technical paper. Cat no 2033055001. 2013. http://www.ausstats.abs.gov.au/ausstats/subscriber.nsf/0/22CEDA8038AF7A0DCA257B3B00116E34/$File/2033.0.55.001%20seifa%202011%20technical%20paper.pdf. Accessed 09 Mar 2016

[24] Australian Bureau of Statistics. 1270.0.55.005–Australian Statistical Geography Standard (ASGS): Volume 5 - Remoteness Structure, July 2011. 2013. http://www.abs.gov.au/ausstats/abs@.nsf/mf/1270.0.55.005?OpenDocument. Accessed 16 Dec 2015

[25] Australian Health Survey 2011–12, Expanded CURF, RADL. Australian Bureau of Statistics, Canberra. 2014. http://abs.gov.au/websitedbs/D3310114.nsf/home/CURF:+Remote+Access+Data+Laboratory+%28RADL%29. Accessed 08 Mar 2016

[26] Landis JR, Koch GG (1977). The measurement of observer agreement for categorical data. Biometrics.

[27] Fortuna RJ, Robbins BW, Halterman JS (2009). Ambulatory care among young adults in the United States. Ann Intern Med.

[28] Booth ML, Bernard D, Quine S, Kang MS, Usherwood T, Alperstein G (2004). Access to health care among Australian adolescents young people’s perspectives and their sociodemographic distribution. J Adolesc Health.

[29] American Heart Association. Atherosclerosis. 2015. http://www.heart.org/HEARTORG/Conditions/Cholesterol/WhyCholesterolMatters/Atherosclerosis_UCM_305564_Article.jsp#.V0a8AORGvwo. Accessed 26 May 2016

[30] Gooding HC, McGinty S, Richmond TK, Gillman MW, Field AE (2014). Hypertension awareness and control among young adults in the National Longitudinal Study of Adolescent Health. J Gen Intern Med.

[31] Johnson HM, Thorpe CT, Bartels CM, Schumacher JR, Palta M, Pandhi N (2014). Undiagnosed hypertension among young adults with regular primary care use. J Hypertens.

[32] Yoon SS, Burt V, Louis T, Carroll MD (2012). Hypertension among adults in the United States, 2009–2010. NCHS data brief.

[33] Kehoe R, Wu SY, Leske MC, Chylack LTJ (1994). Comparing self-reported and physician-reported medical history. Am J Epidemiol.

[34] Wakerman J (2015). Rural and remote health: a progress report. Med J Aust.

[35] Freak-Poli R, Wolfe R, Peeters A (2010). Risk of cardiovascular disease and diabetes in a working population with sedentary occupations. J Occup Environ Med.

[36] Bowlin SJ, Morrill BD, Nafziger AN, Lewis C, Pearson TA (1996). Reliability and changes in validity of self-reported cardiovascular disease risk factors using dual response: the behavioral risk factor survey. J Clin Epidemiol.

[37] White K, Avendano M, Capistrant BD, Moon JR, Liu SY, Glymour MM (2012). Self-reported and measured hypertension among older US- and foreign-born adults. J Immigr Minor Health.

